# State Board of Health

**Published:** 1877-02-20

**Authors:** 


STATE BOARD OF HEALTH.—We learn that the Transactions of the Georgia State Board of Health are in press. We regret that we had not opportunity of examining them before the issue of our present number. Fears are entertained that the Board will be abolished by the present Legislature, as very persistent and formidable opposition has been waged against it since its organization.

WE have received and interesting pamphlet from Prof. A. W. Calhoun, of this city, detailing many operations for cataract. Also copy of remarks before the Medical Journal Association, New York, on *Masturbation and Hysteria* in young children, by Prof. A. Jacobi.

